# Fluoxetine-induced recovery of serotonin and norepinephrine projections in a mouse model of post-stroke depression

**DOI:** 10.1038/s41398-020-01008-9

**Published:** 2020-09-30

**Authors:** Amin Zahrai, Faranak Vahid-Ansari, Mireille Daigle, Paul R. Albert

**Affiliations:** Ottawa Hospital Research Institute (Neuroscience), UOttawa Brain and Mind Research Institute, 451 Smyth Road, Ottawa, ON K1H-8M5 Canada

**Keywords:** Neuroscience, Pharmacology

## Abstract

Chronic treatment with fluoxetine (FLX) is required for its antidepressant effects, but the role of serotonin (5-HT) axonal plasticity in FLX action is unknown. To address this, we examined mice with a stroke in the left medial prefrontal cortex (mPFC) resulting in persistent anxiety-like and depression-like behaviors and memory deficits as a model of post-stroke depression. Chronic treatment with FLX (but not exercise) completely reversed the behavioral phenotype and partially reversed changes in FosB-labeled cells in the mPFC, nucleus accumbens, septum, hippocampus, basolateral amygdala (BLA), and dorsal raphe. In these regions, 5-HT or norepinephrine (NE) innervation was quantified by staining for 5-HT or NE transporters, respectively. 5-HT synapses and synaptic triads were identified as synaptophysin-stained sites on 5-HT axons located proximal to gephyrin-stained or PSD95-stained spines. A week after stroke, 5-HT innervation was greatly reduced at the stroke site (left cingulate gyrus (CG) of the mPFC) and the left BLA. Chronically, 5-HT and NE innervation was reduced at the left CG, nucleus accumbens, and BLA, with no changes in other regions. In these areas, pre-synaptic and post-synaptic 5-HT synapses and triads to inhibitory (gephyrin+) sites were reduced, while 5-HT contacts at excitatory (PSD95+) sites were reduced in the CG and prelimbic mPFC. Chronic FLX, but not exercise, reversed these reductions in 5-HT innervation but incompletely restored NE projections. Changes in 5-HT innervation were verified using YFP staining in mice expressing YFP-tagged channelrhodopsin in 5-HT neurons. Thus, FLX-induced 5-HT axonal neuroplasticity of forebrain projections may help mediate recovery from brain injury.

## Introduction

Major depression is the most prevalent mental illness^1,2^. Post-stroke depression (PSD) occurs within 3 months following stroke and leads to increased mortality and reduced recovery^3–5^. Depression and PSD are treated using first-line antidepressants including selective serotonin (5-HT) reuptake inhibitors (SSRIs)^6,7^. SSRIs augment synaptic 5-HT levels but require several weeks before clinical improvement is seen. This latency may be due to pre-synaptic or post-synaptic adaptations. These adaptations include desensitization of 5-HT1A autoreceptors, enhanced hippocampal neurogenesis, and cortical neuroplasticity^8–10^. However, little is known regarding SSRI actions on cortical neuroplasticity following stroke. Following monocular deprivation in adult rats, chronic SSRI treatment enhances visual plasticity^11–13^. This neuroplasticity occurs via 5-HT signaling to induce neurotrophins and reduce interneuronal inhibition in the visual cortex. Similarly, SSRI treatment of stroke patients increases visual recovery and is associated with reorganization of visual cortex connectivity^14,15^. In some but not all studies, treatment of non-depressed stroke patients with SSRIs improves recovery of motor function^16–18^. In rodents, motor rehabilitation also induces cortical plasticity post-stroke^19,20^ and SSRIs may enhance exercise-induced cortical reorganization and motor learning^21,22^. These studies suggest that SSRIs can induce cortical neuroplasticity following inactivity or injury.

The 5-HT system originates in the midbrain raphe nuclei and projects throughout the brain^23^. The 5-HT projections are unmyelinated axons that contain 5-HT-labeled varicosities along their length. Often these varicosities do not form synapses and mediate diffuse “volume” transmission^24–26^. Research on neurotoxin and brain injury models indicates that 5-HT neurons possess unconventional regrowth properties that require months^27,28^. Several months after trans-section, 5-HT axons can regrow and release 5-HT, which correlates with behavioral recovery^29^. Regrowth of 5-HT axons is also seen after traumatic brain injury^30^. However, the impact of SSRI treatment on 5-HT regeneration has not been addressed. Similarly, little is known regarding plasticity of norepinephrine (NE) projections in adulthood. Like 5-HT projections they recover from neurotoxin injury over time^31^, but are less able to recover after spinal injury^32^. Since SSRIs also indirectly affect the activity of the NE system^33^, chronic SSRI treatment could modulate recovery of NE projections.

In order to address the effect of chronic SSRI treatment on 5-HT and NE neuroplasticity in adulthood, we took advantage of our PSD mouse model^34^. These mice are microinjected with endothelin-1 (ET-1) in the left medial prefrontal cortex (mPFC) to induce a small (~1 mm^3^) unilateral stroke affecting the left infralimbic (IL), prelimbic (PL), and cingulate gyrus (CG) regions. This stroke results in chronic anxiety-like and depression-like behaviors and impaired learning and memory phenotypes. After 6 weeks, the stroke lesion site spontaneously refills with neurons and glia, yet the behavioral and cognitive phenotypes persist. Treatment of PSD mice with chronic fluoxetine (FLX) reversed these phenotypes, while chronic exercise (free running wheel) was ineffective^34,35^. At 6 weeks post-stroke including 2–3 weeks of behavioral testing, there was a global increase in FosB-stained cells at the lesion site and throughout the corticolimbic system^35^. Chronic treatment with FLX for 3 weeks was associated with a reduction in FosB-stained cells towards normal, suggesting a restoration of neuronal activity^35^. We hypothesized that SSRI-induced recovery involves plasticity of 5-HT projections to regions affected by the stroke.

To detect 5-HT axons, we used immunofluorescence for the 5-HT transporter (SERT) or YFP expressed in 5-HT neurons, while NE projections were detected by staining of NE transporters (NET). Confocal microscopic images were reconstructed to quantify the volume of SERT+ or NET+ processes and the density of varicosities in the brains of PSD mice. Co-immunofluorescent labeling was used to detect synapses and triad structures of SERT/synaptophysin+ 5-HT terminals with excitatory (PSD95+) or inhibitory (gephyrin+) synaptic sites^36^. We find that chronic FLX but not exercise, restored 5-HT innervation in the ipsilesional mPFC CG, PL cortex and basolateral amygdala (BLA), and partially restored NE projections to the mPFC. This suggests that chronic SSRI treatment induces recovery of the 5-HT circuitry damaged by the ischemic lesion. These findings highlight a novel role for SSRI-induced neuroplasticity of 5-HT and NE projections in behavioral recovery in a model of PSD.

## Materials and methods

### Mouse cohort and brain sectioning

All studies were approved by the University of Ottawa Animal Care Committee in accordance with guidelines established by the Canadian Council of Animal Care and conformed to ARRIVE guidelines^37^. Histology was performed on brain sections obtained from a behaviorally characterized C57/BL6 mouse cohort (*n* = 32)^35^. To induce ischemia, 11-week-old male C57/BL6 (Charles River Laboratories, Montreal, QC, Canada) or Pet-ChR2 mice (both sexes, see below) weighing 25–28 g at the time of surgery were single-housed on a 12/12 h light/dark cycle with ad libitum access to food and water. After 2 weeks of acclimatization, mice were given consecutive microinjections of 1 μL ET-1 (2 μg/μL = 800 pmol/μL) at two sites in the left mPFC (2): first, AP, 2.0 mm; ML, +0.5 mm; DV, −2.4 mm; second, AP +1.5 mm; ML +0.5 mm; DV, −2.6 mm; sham control mice were injected with vehicle. Ischemic lesions were verified at 4 days post-stroke using magnetic resonance imaging (MRI), and the anxiety phenotype verified at 7 days post-stroke using the elevated plus maze (EPM). PSD mice were randomly divided into three groups of individually housed mice to receive vehicle/fixed wheel (PSD), free running wheel (+Exc) or fluoxetine (+FLX) (80 mg/l to reach 18 mg/kg/day, p.o.), with liquid consumption of 6.5 ± 0.5 ml/day. The automated wheel running distance quantified for 8 mice was 72,660 ± 6280 m/mouse (mean ± SEM) over 3 weeks. Sham mice received vehicle/fixed wheel. After 3 weeks, treatment was maintained and behavioral tests were done (open field (OF), forced swimming test (FST), tail suspension (TS), novelty-suppressed feeding (NSF), a second EPM test, and the Morris Water Maze (MWM) test). Chronic FLX but not exercise alone reversed anxiety, depression, and cognitive phenotypes. The next day, mice had been euthanized, perfused with chilled phosphate-buffered saline (PBS) and fixed with 4% paraformaldehyde by cardiac puncture. Whole brains were isolated, cryo-protected in 20% sucrose, and frozen at –80 °C. 25-μm-thick coronal brain slices were prepared using the coordinates indicated in Table 1^38^. Slices were thaw-mounted on Superfrost slides (Thermo-Fisher, Waltham, MA, USA) and kept at –80 °C. Mice brain sections from different forebrain and midbrain regions (*n* = 3–4) indicated in Table 1 and Supplementary Fig. 1, were stained with antibodies specified in Table 2. A coding system was used so that the performer was blind to the treatment.

**Table 1:** Coordinates relative to Bregma of areas assessed by immunofluorescence.

Brain area	CGctx	PL	IL	NAc	LSN	CA1	CA2/3	DG	BLA	DR
ΔBregma (mm)	1.7	1.7	1.7	1.1	0.5	−1.7	−1.7	−1.7	−2.06	−4.72

Brain areas: CGctx = cingulate cortex; PL = prelimbic; IL = infralimbic; NAc = nucleus accumbens; LSN = lateral septal nucleus; CA1, CA2/3 = hippocampal regions; DG = dentate gyrus; BLA = basolateral amygdala; DR = dorsal raphe.

**Table 2:** Primary/secondary antibodies used for immunofluorescence staining.

	Host	Dilution	Company	Catalog #
*Primary antibody*				
SERT	R	1/1000	Millipore	PC177L
NET	R	1/300	Cedarlane	260003 (SY)
Synaptophysin	M	1/500	Millipore	MAB5258
PSD95	M	1/1000	Abcam	Ab2723
Gephyrin	R	1/1000	Abcam	Ab32206
GFP	C	1/500	Abcam	Ab13970
TPH	S	1/100	Millipore	ab1541
*Secondary antibody*				
G/R 488	–	1/1000	Invitrogen	A11034
G/M 405	–	1/100	ThermoFisher	A-31553
G/M Cy5	–	1/1000	Abcam	AB6563
G/R 555	–	1/1000	ThermoFisher	A-21428
G/C 488	–	1/250	Jackson	103–545–155
D/R 594	–	1/250	Life Technologies	A21207

Species in which antibody was generated/species raised against (secondary antibody): *C* chicken, *D* donkey, *G* goat, *M* mouse, *R* rat, *S* sheep.

### Pet-ChR2 mice and genotyping

Pet-ChR2 mice that express ChR2-YFP exclusively in 5-HT neurons, were generated by crossing Pet-Cre (B6, Cg-Tg(Fev-cre)1ESD/J)^39^ (Jackson Labs, stock number: 012712) with ROSA26-stopflox-ChR2(H134R)-YFP Ai32 mice^40^. For genotyping, DNA was isolated from ear punch tissue sample using solutions from the REDExtract-N-Amp Tissue PCR kit (Millipore Sigma). Genotyping was done using 1X One-Taq Mastermix (New England Biolabs) and 2 pmol of each respective PCR primers: For Pet-Cre (643-bp) 2 pmol of CRE-P1L, 5′-GCC TGC ATT ACC GGT CGA TGC AAC G-3′ and CRE-P2R, 5′-AAA TCC ATC GCT CGA CCA GTT TAG TTA CCC-3′; for ROSA26 (252 bp (ChR2−) and 495 bp (ChR2+)), RR711 (1.6 pmol), 5′-GCA CTT GCT CTC CCA AAG TC-3′, RR712 (1.6 pmol), 5’-GGG CGT ACT TGG CAT ATG AT-3′ and RR713 (0.8 pmol), 5′-CTT TAA GCC TGC CCA GAA GA-3′. Cycling conditions for Pet-Cre were: 2 min at 94 °C; and 12 cycles of 94 °C for 30 s; 68 °C for 30 s, −0.5 °C/cycle; 68 °C for 45 s, followed by 20 cycles of 94 °C for 30 s; 62 °C for 30 s; 68 °C for 45 s; and a final elongation of 5 min at 68 °C. For ROSA26, the cycle was: 2 min at 94 °C, and 33 cycles of 94 °C for 30 s; 64 °C for 30 s; 68 °C for 30 s; and a final elongation of 5 min at 68 °C.

### Immunofluorescent staining

Primary and secondary antibody dilutions were optimized using varying concentrations of each antibody and monitoring the signal-to-noise ratios (Table 2). Sections were incubated at room temperature for 1 h in blocking solution (4% NGS, 1% bovine serum albumin, 0.3% Triton X-100, and 0.05% Tween20 in PBS). Next, sections were incubated with rabbit anti-SERT (1/1000) or rabbit anti-NET (1/300) alone, or in a mixture with mouse anti-PSD95 (1/1000) diluted in blocking solution for 24 h at 4 °C. Sections were then washed 3 × 10 min with blocking solution and incubated for 4 h at room temperature, in goat anti-rabbit-Alexa488 (1/1000) and goat anti-mouse-Cy5 (1/1000) diluted in blocking solution. Sections were then washed 3 × 10 min in blocking solution, 3 × 10 min in PBS, and incubated in a mixture of 10% NRS and 5% NMS diluted in blocking solution for 1 h at room temperature and finally washed 3 × 10 min with blocking solution. Sections were then incubated in goat-anti-mouse (1/100) and goat-anti-rabbit (1/100) monovalent F(ab) antibody fragments diluted in PBS and agitated for 1 h at room temperature. Sections were then washed 3 × 10 min in blocking solution. Sections were then stained for the next set of antibodies. Sections were incubated in a mixture of mouse anti-synaptophysin (1/500) and rabbit anti-gephyrin (1/1000) diluted in blocking solution for 24 h at 4 °C and washed 3 × 10 min in blocking solution. Sections were then incubated in a mixture of goat anti-mouse-Alexa405 (1/100) and goat anti-rabbit-Alexa555 (1/1000) diluted in blocking solution for 4 h at room temperature. Sections were then washed 3 × 10 min in blocking solution and 3 × 10 min in PBS. Coverslips were placed on slides for imaging.

To use YFP as a second marker for 5-HT neurons, Pet-ChR2 mice (*n* = 12) were randomly assigned to sham control (*n* = 3) or ET1 micro-injected stroke (*n* = 9) groups. 4 days post-surgery a subgroup of mice (2 sham, 3 ET1) underwent MRI imaging to visualize the lesion site (Supplementary Fig. 2). To confirm the behavioral phenotype, an EPM test was performed at 1 day pre-stroke and 7 days post-stroke, as described previously^35^. The ET-1-treated stroke mice were further divided into three groups (*n* = 3/group). Randomly, one group was euthanized at 7 days post-stroke, and the rest received vehicle or FLX (18 mg/kg, po) for 3 weeks and were then euthanized, perfused, and sectioned. Sections of Pet-ChR2 brains were incubated with chicken anti-GFP (1:500) and rabbit anti-SERT (1:1000), and with goat anti-chicken-Alexa488 (1:250) and donkey anti-rabbit-Alexa594 (1:250) as secondary antibodies.

### Imaging

Confocal laser scanning images (1024 × 1024 pixels) were acquired on a Zeiss LSM880 AxioObserver Z1 microscope with a Zeiss AxioObserverZ1 mot inverted stand fitted on a IX83 automated inverted platform with a Plan-Apochromat oil-immersed ×63 objective lens (1.4 NA) and solid state lasers (405, 488, 561, and 639 nm) at an exposure of 0.42 μs/pixel and *z* spacings of 0.3 μm (*n* = 3/group). The region of interest template size (µm) was 134.95 × 134.95 × 15.6 (Supplementary Fig. 1). Channels were sequentially scanned to avoid any overlap in the emission/excitation wavelengths (405 + 639, 488, and 561 nm). Images were taken using the ZenBlack 2.3 software. This led to a pixel size of 0.13 μm and a resolution limit of 145 nm according to Abbe’s law^41^. The channels were mostly imaged sequentially using the adjustable emission band path in order to avoid any bleeding through. First, the Alexa 555 was excited by the laser diode 561 nm and emission band path set at: 569–657 nm. Alexa 488 nm was visualized using the Argon laser, 480 nm line, and emission band path: 493–604 nm. Alexa 405 and Alexa 647 were then imaged simultaneously, with the HeNe 633 nm laser and the diode 405 nm laser combined with the emission band path set at 410–501 and 638–745 nm, respectively. To compensate for light scattering and the point spread function, acquired images were deconvolved using AutoQuant X3.1 software with 10 iterations, high noise level, adaptive point spread function (PSF), theoretical PSF, and a refractive index value for the objective lens’ immersion medium of 1.515. A correction for the distance from the coverslip was also applied where the length of the unblurry region of the *Y*–*Z* plane was measured (4–8 μm) and entered for three-dimensional deconvolution. To maintain the *x*/*y*/*z* voxel size, each deconvolved image was saved as an Imaris file (Bitplane IMS 5 file).

### Image analysis using Imaris x64 9.1.2

Analysis with Imaris was done as previously described^42–44^. SERT, NET, or YFP immunolabeled fibers were reconstructed in 3D with Imaris’s surface rendering function and axonal volume density was calculated. Imaris’s masking function was then used to remove intra-fiber labeling and conserve the fluorescently tagged synaptophysin outside of SERT + axons (Syn^out^), gephyrin (Geph^out^), and PSD95 (PSD95^out^) located outside of the SERT surfaces made previously. This was then used to mark putative inhibitory (gephyrin) and excitatory (PSD95) sites. Also, removing synaptophysin’s fluorescence signal outside of the created SERT surfaces ensured that only synaptophysin within the SERT+ fibers were being identified, and putative serotonergic synaptic boutons (SYN^SERT+^) were then quantified. Imaris’s spot detection tool was then applied to each of the constructed masks (serotonergic boutons as Syn^SERT+^, contacts with inhibitory synapses as Syn^SERT+^/Geph^out^, and with excitatory synapses as Syn^SERT+^/PSD95^out^) for detection of spots with a diameter of 0.6 μm and above. This size was selected based on the Z step-size to ensure that spots were present in a minimum of two confocal optical slices, and that spots are present in 3D space and are not just artifacts from Imaris, as previously described^43,44^. Imaris’s spot colocalization tool was then used to label either Syn^SERT+^/Geph^out^ or Syn^SERT+^/PSD95^out^ spot pairs, within a distance of 0.6 μm between spots within a pair, to identify pairs of putative 5-HT contacts with inhibitory or excitatory synapses, respectively. Syn^SERT+^ boutons that were located within 0.6 μm of Syn^out^/Geph^out^ (5-HT synapse with inhibitory synapse) or Syn^out^/PSD95^out^ (5-HT synapse with excitatory synapse) spots pairs were also labeled as serotonergic inhibitory and excitatory triads, respectively^36^. SERT+ fibers were reconstructed using Imaris’s filament tool, with the filament diameter being 0.5–0.6 μm, and the axonal volumes were determined using surface reconstruction of SERT+ axons. A minimal ratio of branch length to trunk radius of 1.5–2.5 to reduce background signal, with further filter processing was done to remove artificial fibers. The number of SERT+ varicosities was determined using the spot detection tool in Imaris, with spots of varying sizes fitted into corresponding varicosities.

### Statistical analysis

Quantification of data was blinded. The data were plotted and analyzed using GraphPad Prism 8.0 software (GraphPad Software, La Jolla, CA, USA; www.graphpad.com). Data are expressed as mean ± SEM. Images from 3 to 4 randomly selected mouse brains were averaged for each brain region, based on our previous study^35^. Comparison of separate pairs of data was done using *t*-test. One-way ANOVA analysis of variance was performed for comparing multiple groups such as sham, PSD, +Exc, and +FLX groups, with significance being set at *p* < 0.05. Post-hoc comparisons were done using Tukey’s multiple comparisons test.

## Results

**Fig. 1:**
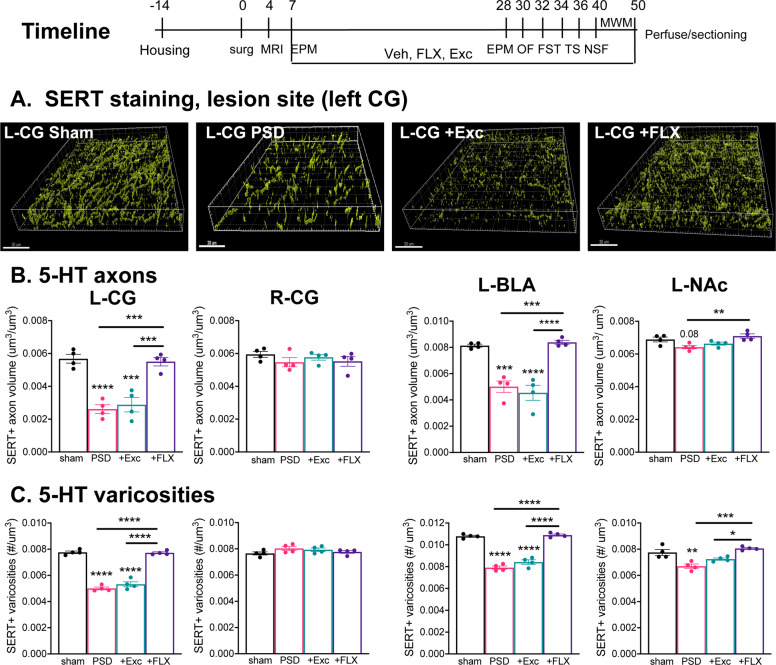
Chronic FLX but not exercise reverses stroke-induced loss of 5-HT projections. Brain tissues from our previous study^35^ were used. Timeline (days): Male C57BL6 mice were individually housed (Housing), microinjected at 11 weeks old with vehicle (Sham-Ctrl) or ET-1 (PSD) in the left mPFC (surg); lesions were verified (MRI); and anxiety like phenotype assessed using the elevated plus maze test (EPM). Then the PSD mice were treated with free running wheel (+Exc) or fluoxetine (+FLX), with fixed wheel and vehicle (Veh) as control (PSD) and compared to Sham-ctrl. Behavioral assays included EPM, open field (OF), forced swim (FST), tail suspension (TS), and novelty suppressed feeding tests (NSF) followed by the Morris water maze spatial memory test (MWM). The mice were then sacrificed (day 50), perfused, and brains recovered, sectioned, and immunofluorescence done on a randomly chosen subset of 4 brains/group as presented below. The PSD mice displayed increase anxiety-like and depression-like behaviors and impaired spatial memory; these phenotypes were reversed with FLX, but not Exc treatment. **a** Chronic FLX but not exercise (Exc) reverses the reduction in 5-HT projections in the lesion site at 6 weeks post-stroke. Shown are representative reconstructed flattened confocal images of SERT-stained sections of the ipsilesional (left, L) cingulate gyrus (CG) at ×63 magnification. Scale bar, 10 μm. **b** Quantification of total SERT+ axonal volume normalized to tissue volume. **c** Quantification of 5-HT varicosity number normalized to tissue volume. Quantification is shown for the ipsilesional (left, L) and contralesional (right, R) CG, left basolateral amygdala (L-BLA) and left nucleus accumbens (L-NAc) of Sham control, PSD, PSD+Exc, and PSD+FLX mice (*n* = 4). See Supplementary Fig. 3A for additional right brain data. Data are shown as mean ± SEM; *p < 0.05, **p < 0.01, ***p < 0.001, ****p < 0.0001 compared to sham or as indicated; one-way analysis of variance (Tukey’s post-hoc).

In this study, we initially examined brain tissues from our previous study of PSD mice treated according to the timeline shown in Fig. 1^35^. Following individual housing, they received unilateral microinjection of ET-1 in the left mPFC, resulting in a small 1-mm^3^ stroke confined to the mPFC as assessed using cresyl violet staining^34^. The behavioral phenotype of the mice was assessed at 1 week post-stroke using the EPM test, and then the mice were treated with vehicle, FLX or exercise (Exc, free running wheel), and subjected to a battery of behavioral tests over the next 4 weeks, sacrificed and brains harvested. Exercise-treated PSD mice showed little or no behavioral improvement compared to vehicle, while those treated with FLX completely recovered to sham control levels. The PSD mice showed evidence of a widespread dys-regulation of brain activity, with changes in the numbers of FosB+ cells that were partially reversed by FLX but not exercise treatment. To address whether 5-HT projections were also affected in these areas, SERT immunofluorescence was used to detect 5-HT axons and varicosities on the left (lesioned) and right sides of the brain (Fig. 1). The volume of SERT+ processes and the density of SERT+ varicosities were measured in a defined volume in the mPFC (CG, PL, and IL), nucleus accumbens core (NAc), BLA, lateral septum (LS), hippocampus (CA1, CA2/3, and dentate gyrus—DG) and the dorsal raphe nucleus (DR) (Table 1, Supplementary Fig. 1).

As shown in Fig. 1a, there was a striking reduction in SERT+ fibers in the left mPFC of PSD mice that was reversed by FLX treatment. In the left CG of PSD mice a significant 50% reduction in the density of SERT+ axonal volume and varicosity density was seen compared to sham mice (Fig. 1b, c). While chronic exercise (+Exc) did not alter this deficiency, a full recovery of both SERT+ axonal volume and varicosity density was obtained upon chronic FLX treatment (Fig. 1). Similarly, after stroke the left PL showed reduced SERT+ varicosity density (but normal axonal volume density) and FLX (but not Exc) induced a complete recovery (not shown). By contrast, the IL (not shown) and the non-lesioned right CG (Fig. 1) showed no deficits in SERT+ axons or varicosities.

Surprisingly, deficits in 5-HT innervation following stroke were also seen in other brain regions. Most strikingly, SERT+ axonal volume and varicosity density were reduced by 50% in the left BLA of PSD mice, and this was rescued by chronic FLX but not exercise treatment (Fig. 1b, c). Similarly, in the left NAc SERT+ varicosity density was reduced with a trend reduction in axonal volume and these recovered to sham level after FLX treatment (Fig. 1), with no changes seen in the non-lesioned right BLA and NAc (Supplementary Fig. 3A). Conversely, the serotonergic DR of PSD mice did not show changes in SERT+ process volume or varicosity density, without or with treatment (not shown). Other limbic regions, including hippocampal regions and septum, did not show significant changes in the volume of their serotonergic axons. Interestingly, the density of SERT+ axons and varicosities in intact (right side, sham) brain was greatest in the BLA and DR and lowest in the hippocampal DG (Supplementary Fig. 4). To address whether 5-HT release sites are affected, SERT/synaptophysin co-stained sites were quantified in selected brain regions. In the left CG and BLA, stroke reduced SERT+ bouton density and this was rescued upon chronic FLX but not Exc treatment (not shown). No changes were seen in PL, IL, or DR. Our previous study showed a full recovery of anxiety-like and depression-like behaviors induced by FLX following ischemia in the left mPFC in these mice^35^. The present post-mortem analysis of their tissues shows that this behavioral recovery is associated with a recovery of 5-HT projections, particularly at the lesion site (CG and PL) and in the left BLA and NAc.

**Fig. 2:**
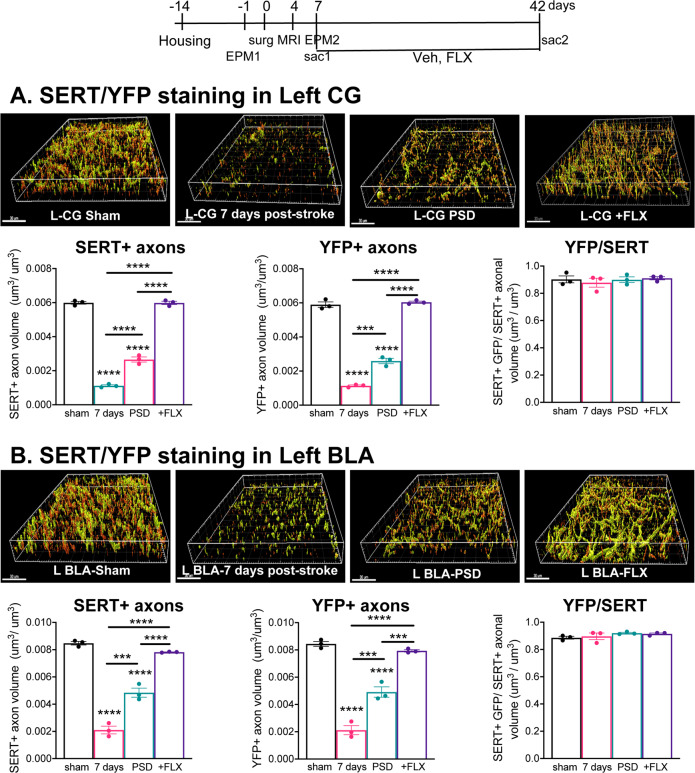
Chronic FLX induces recovery of ChR2-YFP-labeled 5-HT projections in PSD mice. Timeline (days): Pet-ChR2 mice expressing ChR2-YFP only in 5-HT neurons were single housed (Housing), given stroke (*n* = 9) or sham (*n* = 3) surgery in the left mPFC at day 0 (surg), as done previously (Fig. 1). The mice were examined by 7 T MRI (day 4) and EPM tests before and after stroke (EPM1, EPM2) to verify lesion site and anxiety behavior, an early marker of the PSD phenotype in this model (Supplementary Fig. 2). Three of the PSD mice were sacrificed 6 h after EPM2 (7 day, sac1). The remaining sham and stroke mice were treated with vehicle (drinking water), or half of the stroke mice with 18 mg/kg/day FLX in drinking water (+FLX) for 6 weeks post stroke and sacrificed (sac2). Representative reconstructed ×63 confocal images of left cingulate gyrus of mPFC (L CG) **a** and L BLA **b** are shown above, with quantification of left and right sides shown below. Brain sections from sham control mice, 7 days post stroke, PSD and PSD + FLX were co-stained for SERT (red) and YFP (green) to visualize SERT + ChR2-YFP-expressing 5-HT projections. At one week post-stroke there was a loss of both SERT and YFP staining in the left CG **a** and left BLA **b** compared to the sham ctrl, with no changes in the right brain regions (see Supplementary Fig. 3B). After 6 weeks, the reduction persisted (PSD) with a complete recovery of SERT-positive and YFP-positive projections after FLX (+FLX) in left CG and BLA compared to sham. Colocalization of YFP/SERT staining was reconstructed using Imaris software and was extensive with a ratio of SERT/YFP+ to total SERT+ processes of 0.9 that did not differ among groups. Size bar, 30 µm. Bars represent mean ± SEM (*n* = 3), ****p < 0.0001 compared to sham or as indicated; one-way analysis of variance (Tukey’s post-hoc).

To address whether changes in SERT+ immunostaining reflect altered 5-HT projections rather than SERT expression, we used channelrhodopsin-2-YFP (ChR2-YFP) as a second membrane-bound marker for 5-HT projections. We generated Pet-ChR2 mice and verified in the DR that ChR2-YFP is colocalized with tryptophan hydroxylase (TPH) in nearly all 5-HT neurons (Supplementary Fig. 2A), as reported previously^45^. These mice were given ET-1-induced stroke in the left mPFC (Timeline, Fig. 2) and in vivo MRI was done 4 days post stroke to verify correct lesion size and location in the left mPFC in mice with stroke vs. sham surgery (Supplementary Fig. 2B). In the EPM test, a sensitive marker of the PSD phenotype^34,35^, the stroke mice showed increased anxiety-like behavior 1 week post stroke compared to before stroke (Supplementary Fig. 2C). The PSD mice were then treated with vehicle or 18 mg/kg/day FLX for 5 weeks, sacrificed and brain sections co-stained for YFP and SERT (Fig. 2). Importantly, YFP and SERT staining was 90% colocalized, indicating that both markers detect 5-HT processes. One week post stroke both YFP and SERT staining were strongly reduced in the left CG and BLA compared to the sham control. After 6 weeks (PSD group), the reduction partly recovered but persisted in both regions, but was reversed by chronic FLX treatment. In the right CG but not BLA, a slight but significant reduction in YFP+ projections was seen only in the PSD group, suggesting that the stroke may affect a subset right side 5-HT projections (Supplementary Fig. 3B). Thus, chronic FLX enhances recovery of 5-HT projections to the lesion site and the BLA.

**Fig. 3:**
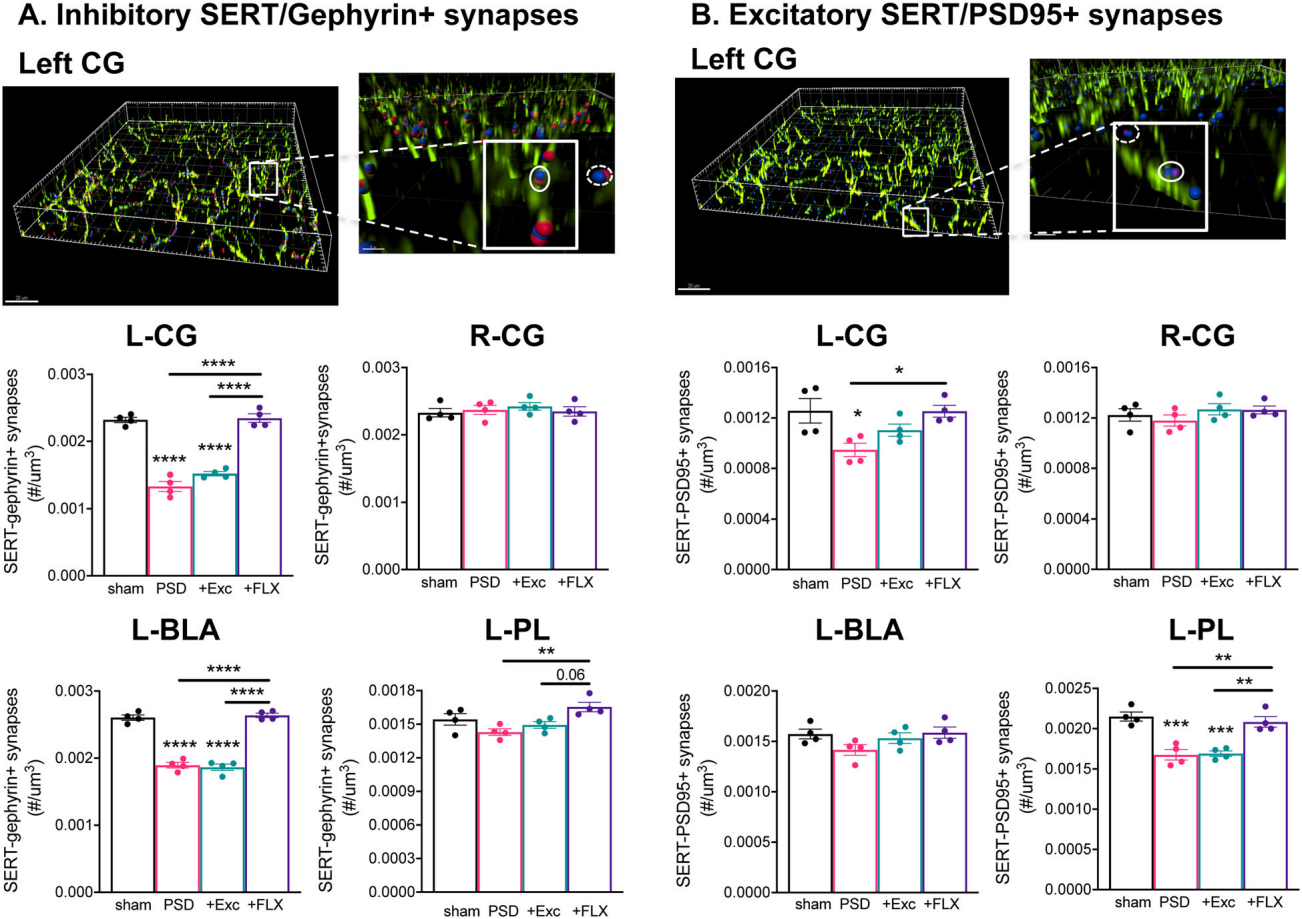
Chronic FLX but not exercise reverses stroke-induced reductions in 5-HT synapses. Above: representative reconstructed ×63 confocal images of left mPFC (CG) from sham control co-stained for SERT (green), synaptophysin (blue), and gephryn (red) or PSD95 (purple) to visualize 5-HT synapses with inhibitory and excitatory synapses (within 0.6 µm, circled), respectively, distinct from non-5-HT synapses (dashed circles); synaptophysin, gephyrin, and PSD95 boutons are reconstructed using Imaris software. Size bars, 20 and 8 µm. Below: 5-HT synapses to excitatory and inhibitory synapses were quantified in both ipsilesional left and contralesional right CG, left BLA and left PL of sham, or stroke (PSD), exercise- (+Exc) or FLX-treated (+FLX) stroke mice. **a** Quantification of 5-HT synapses to inhibitory synapses. Sections were co-stained for SERT, synaptophysin and gephyrin; SERT/synaptophysin+ boutons within 0.6 μm of gephyrin+ puncta were quantified and normalized to tissue volume. **b** Quantification of 5-HT synapses to excitatory synapses. Sections were co-stained for SERT, synaptophysin and PSD95: SERT/synaptophysin+ boutons within 0.6 μm of PSD95+ puncta were quantified and normalized to tissue volume. Data for right BLA and PL are in Supplementary Fig. 6A. Bars represent mean ± SEM (*n* = 4). *p < 0.05, **p < 0.01, ***p < 0.001, ****p < 0.0001; one-way analysis of variance (Tukey’s post-hoc).

**Fig. 4:**
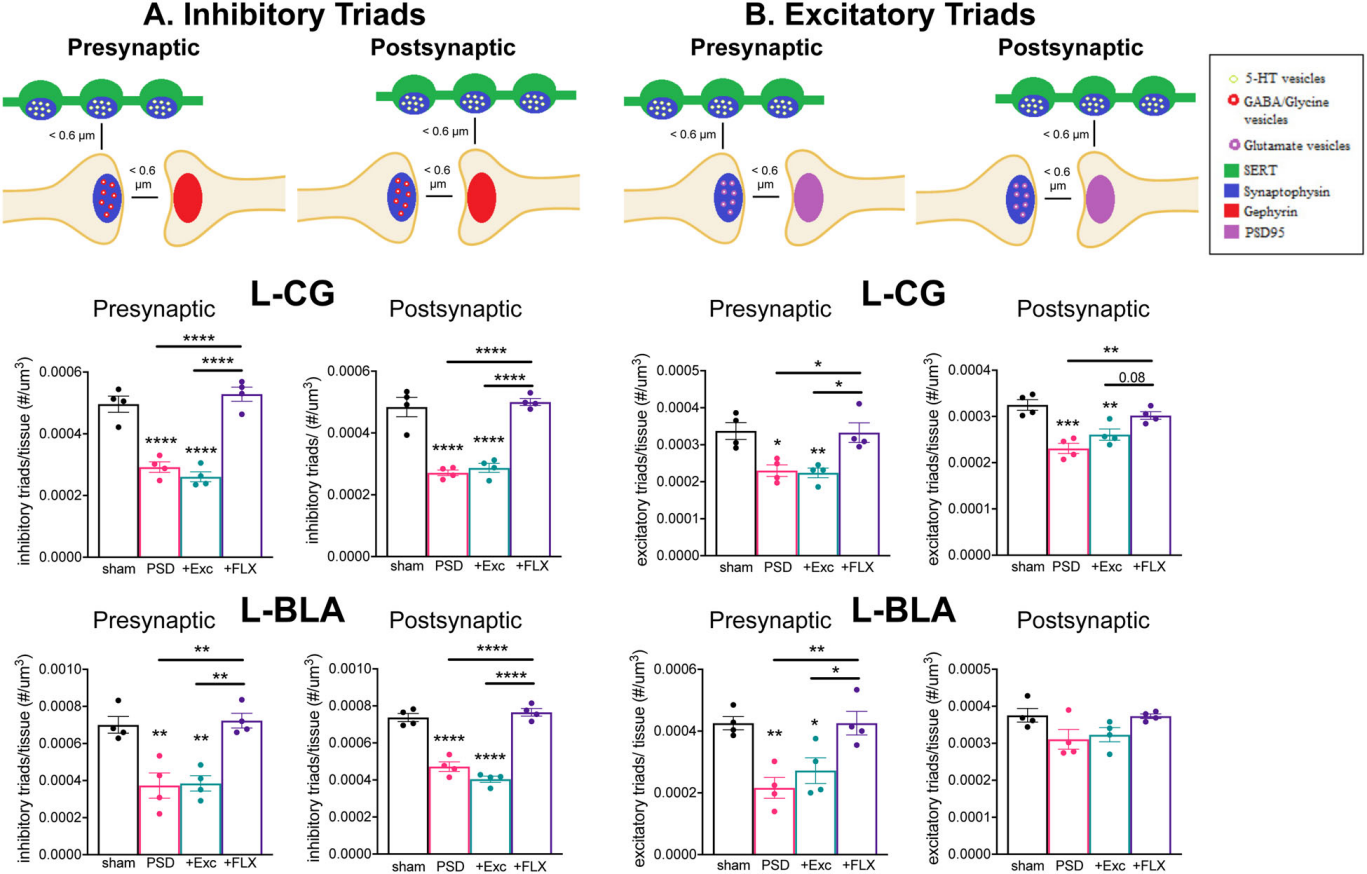
Chronic FLX but not exercise reverses stroke-induced reductions in excitatory and inhibitory 5-HT triadic synapses. Sections were stained for SERT, synaptophysin and either gephyrin (5-HT inhibitory triads) or PSD95 (5-HT excitatory triads). Above: models showing inhibitory or excitatory pre- and post-synaptic triads. Below: quantification of 5-HT triads is shown for ipsilesional left CG and BLA in sham, or stroke (PSD), exercise- (PSD + Exc) or FLX-treated (PSD + FLX) stroke (see Supplementary Fig. 6B for right CG, BLA). **a** Inhibitory 5-HT Triads. Synaptophysin/SERT+ boutons within 0.6 μm of inhibitory (SERT−/synaptophysin+ boutons within 0.6 µm of gephyrin+ puncta) synapses were quantified and normalized to tissue volume. **b** Excitatory 5-HT Triads. Synaptophysin+ boutons within SERT+ fibers located within a distance of 0.6 μm of excitatory (SERT−/synaptophysin+ boutons within 0.6 µm of PSD95+ puncta) were quantified and normalized to tissue volume. Bars represent mean ± SEM (*n* = 4). *p < 0.05, **p < 0.01, ***p < 0.001, ****p < 0.0001; One-way analysis of variance (Tukey’s post-hoc).

In our previous study, chronic FLX treatment of PSD mice had differential actions on inhibitory and excitatory neuronal activity especially in the mPFC, as measured by the number of FosB+ cells^35^. Thus, we quantified 5-HT synapses to inhibitory and excitatory synaptic sites, as well as triadic synapses^36^ in stroke-affected regions including mPFC (CG, PL, IL), BLA and DR (Figs. 3 and 4). SERT-synaptophysin co-immunofluorescence was used to label 5-HT presynaptic sites, while proximal (<0.6 µm) PSD95-labeled or gephyrin-labeled post-synaptic sites were quantified to assess 5-HT synapses with inhibitory or excitatory synaptic sites, respectively. In addition, sites with synaptophysin/SERT+ axons in close proximity (<0.6 µm) to synaptophysin and either gephyrin or PSD95, were used to define inhibitory and excitatory triads, respectively (Fig. 4). In the left CG and BLA, PSD mice showed a strong reduction in 5-HT contacts with inhibitory synapses that was reversed by chronic FLX but not exercise (Fig. 3a). A similar, but weaker change in excitatory synapse density was seen in left CG but not BLA (Fig. 3b). In contrast, in the PL there was a marked reduction in 5-HT contacts at excitatory synapses that was reversed upon chronic FLX treatment. Interestingly, 5-HT synapses to inhibitory vs. excitatory synapses were more prevalent in the CG and BLA, but the opposite was seen in PL and DR (Supplementary Fig. 5). No significant effect of stroke or FLX treatment was seen in 5-HT inhibitory or excitatory synaptic contacts in the right CG (Fig. 3), BLA or PL (Supplementary Fig. 6A), or IL and DR (not shown). Thus, the ischemic lesion resulted in region-specific alterations in 5-HT input to inhibitory or excitatory synapses persisting for at least 6 weeks that were reversed by chronic FLX treatment. To further probe synaptic plasticity in the PSD mice, we examined 5-HT synaptic triads located pre- and post-synaptically (Fig. 4). Again, in CG and BLA, both pre-synaptic and post-synaptic inhibitory triads were strongly reduced in PSD mice, and they recovered upon FLX treatment (Fig. 4a). Interestingly, similar reductions were seen in excitatory triads (pre- and post-synaptic in CG; pre-synaptic in BLA), which were reversed upon FLX but not Exc treatment (Fig. 4b). No changes were seen in right side regions (Supplementary Fig. 6B). These results indicate that synapse-specific modulation of inhibitory vs. excitatory triads occurs on the lesioned side following ipsilateral ischemia and is reversed by FLX treatment.

**Fig. 5:**
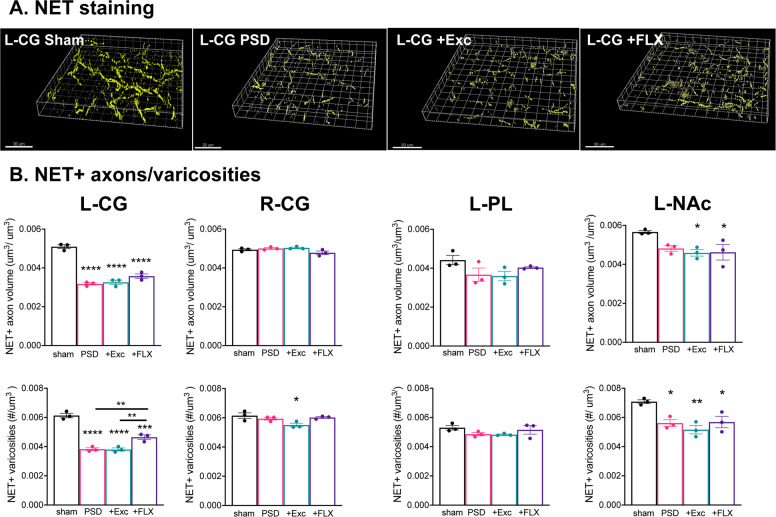
Loss of norepinephrine projections after stroke is partially restored by chronic FLX treatment. **a** Chronic FLX incompletely reverses the reduction in NE projections at the lesion site at 6 weeks post-stroke. Shown are representative reconstructed confocal images of NET-stained sections of the ipsilesional (left, L) cingulate gyrus (CG), prelimbic cortex (PL), and nucleus accumbens (NAc) of sham, post-stroke (PSD), and chronic FLX-treated post-stroke (FLX) mice (×63 magnification). Scale bar is 30 μm. **b** Quantification of total NET+ axonal volume normalized to tissue volume (above) and of NET+ varicosity number normalized to tissue volume (below). Quantifications represent the left and right CG, left PL and left NAc of Sham control, or vehicle-treated (PSD), exercise-treated (+Exc) or FLX-treated (+FLX) stroke mice (*n* = 3), shown as mean ± SEM. See Supplementary Fig. 6C for right PL and NAc data. *p < 0.05, **p < 0.01, ***p < 0.001, ****p < 0.0001; one-way analysis of variance (Tukey’s post-hoc).

Since the NE system strongly projects to the mPFC and is implicated in antidepressant action, we also examined NE projections labeled using NET staining (Fig. 5a). In sham mice, the density of NET+ axonal volume and varicosities was greatest in the DR and BLA and lowest in the hippocampal DG, compared to most regions including the CG (Supplementary Fig. 4). In the PSD mice, NE projections to the ipsilesional left (but not right) CG were reduced compared to sham control, and this was not reversed by FLX or exercise (Fig. 5b). The left NAc in exercise-treated or FLX-treated PSD mice showed reductions in NET+ axonal volume density compared to sham, with no change in the PL. The density of NET+ varicosities was also reduced in PSD mice in the left CG and NAc compared to sham, and FLX produced a partial reversal only in the left CG. In PSD mice, no changes in NET staining were seen in right side regions except in the right NAc. In the right NAc stroke alone had no effect, but exercise reduced NET+ axonal density while FLX reduced NET+ varicosity density, compared to sham (Supplementary Fig. 6C). No changes were seen in other brain regions, including in the BLA in which 5-HT projections were reduced, indicating different sensitivities of 5-HT and NE projection networks. Thus, ischemic lesion reduces NE innervation to different regions than 5-HT, and chronic FLX was ineffective to enhance recovery of NE projections except for a partial effect on NE varicosities at the site of ischemia (left CG).

## Discussion

The plasticity of the 5-HT system has been extensively studied in development^46,47^, but less so in adulthood. Studies in rodents have shown that unlike other neurons, 5-HT neurons can regrow following injury in adulthood. Following traumatic brain injury, 5-HT axons spontaneously regrow over a period of months into target tissues^27,29,30^. Sprouting of 5-HT axons has been shown in neurotoxin lesion models^27,28^, sometimes with negative effects as in Parkinson disease models^48^. Using our mouse model of PSD we addressed whether 5-HT axonal regrowth occurs in stroke-damaged tissue and whether antidepressant treatments might enhance this regrowth. We find a partial spontaneous regrowth of 5-HT axons into the lesioned left mPFC at 6 weeks post-stroke, with no change or reduced innervation (in right CG) on the right side. 5-HT innervation of IL appeared normal while it was slightly impaired in the PL, with the greatest impairment in CG, consistent with the pattern of 5-HT regrowth from the median forebrain bundle^29^. However, this spontaneous regrowth was insufficient to improve the behavioral phenotype. In contrast, chronic FLX treatment restored the loss of 5-HT innervation in the mPFC, while exercise was ineffective^35^. This correlated with the effect of FLX but not exercise to restore the behavioral and cognitive impairments in these mice. Furthermore, FLX treatment restored 5-HT innervation at sites distal from the stroke, like the BLA. FLX-induced recovery of SERT-stained processes did not appear to be an effect on SERT expression, since we saw the same changes in 5-HT innervation using ChR2-YFP as a marker. In fact, chronic SSRI treatment tends to down-regulate SERT expression^49,50^. Thus, FLX-induced behavioral recovery in the PSD mouse is associated with a global regrowth of 5-HT projections. These projections appear to make region-specific synaptic contacts. For example, the stroke reduced 5-HT synapses and triads to inhibitory spines in the CG and BLA and at excitatory contacts in the PL and less strongly in CG and BLA (triads only). All of these changes were reversed by FLX treatment. However, since both GABAergic interneurons and glutamatergic neurons receive excitatory (PSD95+) and GABA (Gephyrin+) synapses the identity of the neurons innervated by 5-HT projection is not identified using our method. Nevertheless, this specificity mirrors FLX-induced restoration of inhibitory–excitatory balance in the mPFC^35^ and argues for a functional role for these contacts in behavioral recovery^22^.

Interestingly, there was a marked deficiency of 5-HT innervation in the left BLA of PSD mice, while other projection sites appeared to retain normal 5-HT innervation. The BLA is located closer to the raphe nuclei than the ischemic site in the left mPFC, suggesting that the axonal damage may have caused retraction of collateral branches. Single cell tracing of vGLUT3+ 5-HT neurons show collaterals to amygdala, striatum, and the prefrontal cortex^51^. Using virally mediated GFP labeling in SERT-Cre mice, strong projections from the ventral dorsal raphe (B7v) to both the amygdala and mPFC were identified^52^, although retrograde tracing reveals that distinct raphe 5-HT neurons form synapses at mPFC and BLA^53^. Global mapping of 5-HT projections using retrograde tracing and Clarity protocols has revealed distinct 5-HT projections to cortical and amygdala regions^54^. Stimulating the cortical projections to the raphe reduces behavioral despair, while activating the amygdala projections drives anxiety. Our results show that 5-HT projections to the mPFC and BLA are both sensitive to unilateral ischemia of the mPFC and respond to chronic FLX, suggesting a connection between the two. Chronic FLX not only increased varicosity number but also restored 5-HT synapses, especially inhibitory contacts in these regions. Hence, FLX may mediate behavioral recovery primarily by restoring 5-HT regulation of GABAergic transmission in the PFC^13,55,56^. Consistent with this, chronic FLX treatment appears to preferentially activate fast-spiking interneurons, with little effect on pyramidal activity^57^.

Increasing research implicates SSRI-induced neuroplasticity of injured neural circuits in enhancing recovery from stroke^58^. Without FLX treatment, 5-HT neurons show enhanced sprouting in response to electro-coagulation injury, in part due to their insensitivity to axonal growth inhibitors^27^. In a Parkinson’s disease model, aberrant sprouting of striatal 5-HT projections was seen upon treatment with l-DOPA^48^. In agreement with our findings, chronic (4-wk) FLX but not desipramine treatment of normal adult rats increases SERT+ axons and their branching in piriform cortex and NAc, although behavioral improvement was not tested^59^. Oppositely, in normal adult mice chronic FLX reduced the density of hippocampal 5-HT innervation^60^. Interestingly, we did not see changes in 5-HT projections in these regions in our mouse PSD model^35^.

Reductions in 5-HT neurons and their projections are seen in other models of depression and in humans.^11,61,62^ In post-mortem studies, reductions in 5-HT innervation of the orbital frontal cortex^63^, and in the number of 5-HT neurons^64^ were seen in depressed compared to control brain samples. Rodent models of depression, including chronic unpredictable stressed rats or Flinders sensitive line of “depressed” rats, also have reductions in 5-HT neurons and 5-HT projections to the mPFC^65,66^. On the other hand, chronic deep brain stimulation of the mPFC increased the size and density of 5-HT terminals in the mPFC and hippocampal DG of mice subjected to chronic social defeat stress^67^. Increased axonal outgrowth is seen in pluripotent cells from SSRI responsive vs. resistant depressed patients differentiated to a 5-HT-neuron-like state^68^. These results implicate the neuroplasticity of 5-HT projections in mediating effective antidepressant treatments. In combination with the ability of FLX to enhance neuroplasticity of post-synaptic cortical neurons^8,69–71^, these presynaptic actions on 5-HT projections may lead to functional 5-HT synapses and restore behavioral and cognitive functions.

Neuroplasticity of neural projections following chronic SSRI treatment of PSD mice was not limited to 5-HT innervation. As shown for 5-HT, NET+ processes, and varicosities were reduced at 6 weeks post stroke, especially in the left CG. These findings provide the first evidence that like 5-HT, the NE system can restore projections lost following ischemic damage. Although chronic FLX treatment had little effect on NE axonal recovery, NET+ varicosity density was partially recovered in the CG. However in the NAc, NE axonal, and varicosity density did not recover. The NE system integrates inputs from many brain regions and also projects widely to these brain regions^72^. The presence of NE projections facilitates regrowth of 5-HT processes following neurotoxin treatment^73–75^. Antidepressant-induced crosstalk between the 5-HT and NE systems^33^ or^49^ the uptake and release of 5-HT from NE neurons^76,77^ (as seen in dopamine neurons^78^) could mediate the effect of chronic FLX on NE projections at the lesion site, but this remains to be tested^77,79^.

In conclusion, we find that unilateral ischemic lesion of the mPFC reduces 5-HT and NE innervation of the lesion site, but also at other brain regions distal from the stroke. Chronic FLX-induced behavioral and cognitive recovery following ischemia was associated with 5-HT and NE axonal plasticity in these regions. Whether similar mechanisms are induced by chronic FLX in other non-injury forms of depression associated with stress or genetic risk factors remains to be examined^80,81^. In major depression and mild cognitive impairment, deficits in gray matter volume and 5-HT innervation are seen in the PFC^82,83^, and reduced cortical thickness has been associated with 5-HT genetic markers^84,85^. The finding that effective treatment of major depression is associated with a reversal of reduced cortical thickness^86^ and increased connectivity to the 5-HT system^87^^,88^ suggests that SSRI-induced axonal neuroplasticity may be an important mechanism in its antidepressant actions in humans^8,71^.

## Supplementary information

Supplemental Figures
