# Supplementary material for: Fluoxetine-induced recovery of serotonin and norepinephrine projections in a mouse model of post-stroke depression
Source: Transl Psychiatry. 2020 Sep 30;10:334. doi: 10.1038/s41398-020-01008-9 (PMC7527452; doi:10.1038/s41398-020-01008-9)
Supplement: Supplementary file 1 — Supplemental Figures [file 41398_2020_1008_MOESM1_ESM.pdf]

Supplemental Figure 1

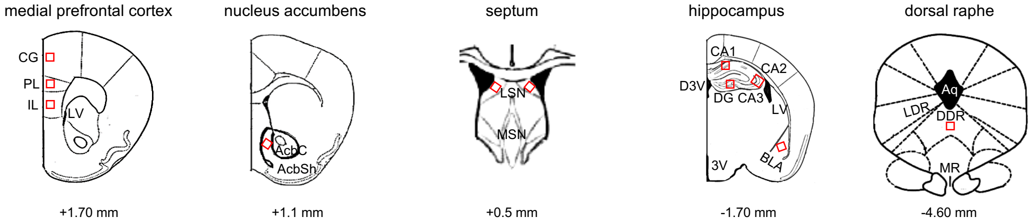

Supplemental Fig. 1. Regions of interest (ROIs) for immunofluorescence.

Coronal brain slices were taken using a cryostat with respect to Bregma (distance shown below) to match sections across groups. ROIs were 134.95 x 134.95 x 15.6 µm and are indicated by red squares. ROIs were located in the mPFC (infralimbic (IL), prelimbic (PL), and cingulate gyrus (CG)), nucleus accumbens core (AcbC), basolateral amygdala (BLA), lateral septum (LSN), hippocampus (CA1, CA2/3, and dentate gyrus-DG) and the dorsal dorsal raphe nucleus (DDR). Also shown are the lateral ventricle (LV), nucleus accumbens shell (AcbSh), medial septum (MSN), third ventricle (3V), dorsal 3V (D3V), median raphe (MR) lateral DR (LDR) and aqueduct (Aq). Adapted from the Mouse Brain Atlas.

Supplemental Figure 2

A. ChR2-YFP/TPH co-staining in dorsal DR

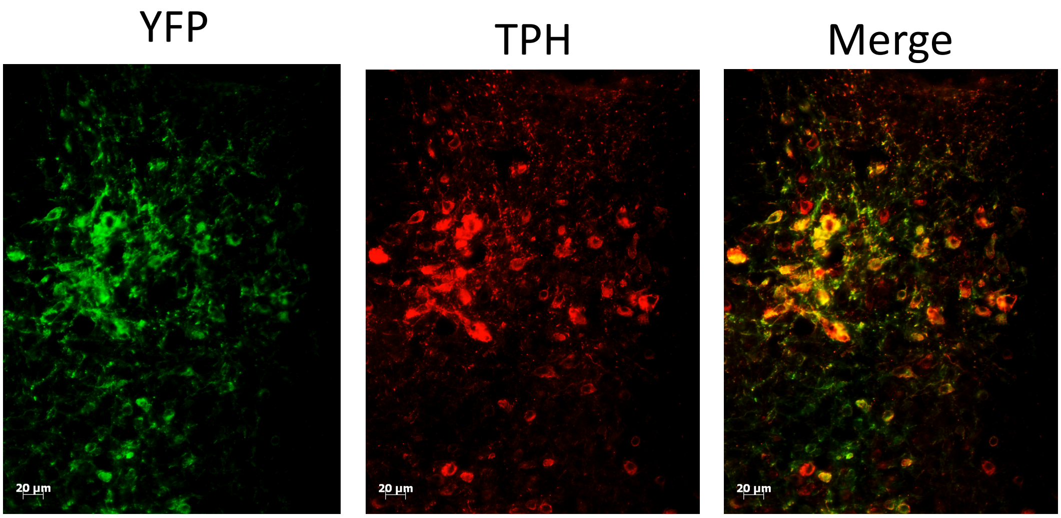

B. MRI-4 days post-surgery

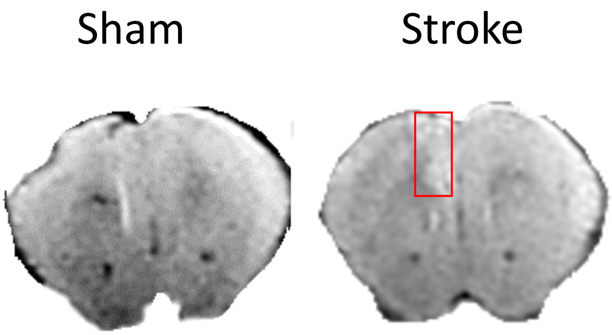

C. EPM Pre-/Post-stroke

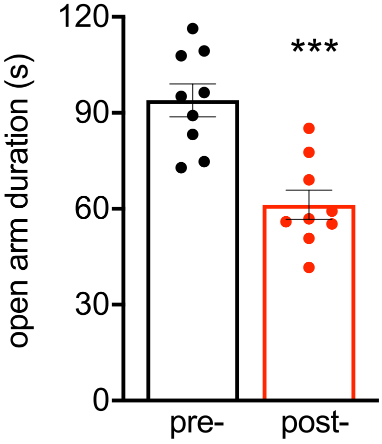

Supplemental Figure 2. Characterization and ET-1-induced lesion in mPFC of Pet-ChR2 mice

A) Co-staining of ChR2-YFP and TPH in the DR of Pet-ChR2 mice. A representative section from the dorsal DR region (Table 1) from normal adult Pet-ChR2 mouse was stained for yellow fluorescent protein (YFP, green) and tryptophan hydroxylase (TPH, red), a marker of 5-HT neurons (see Table 2 for antibodies). The merge shows an almost complete co-localization (yellow). Images were acquired on a Zeiss Axio Observer D1 microscope under 20X magnification and analyzed using Axiovision imaging software (RRID:SCR_002677). Size bar as shown.
B) ET-1 lesion site in Pet1-ChR2 mice visualized *in vivo* by MRI. See **Fig. 2** for timeline. Pet-ChR2 mice were microinjected with vehicle (sham ctrl, n=3) or endothelin 1 (ET-1, n=9) in the left medial prefrontal cortex (mPFC). 4 days post stroke, lesion verified by using MRI. Shown are representative 7-T MRI images in sham and stroke brain at the mPFC. At 4 days poststroke, lesions in the left mPFC (red box) were verified by magnetic resonance imaging (MRI). Briefly, using a 7Tesla GE/Agilent MRI machine, a subset of animals (n=5 total: sham, n=2; stroke, n=3) was anesthetized with isoflurane in O_2: induction at 3%, maintenance at 1.5%. Serial MRI images were taken from prefrontal cortex at 300 μm thickness. A fast spin echo pulse sequence was used with repetition time = 4500 ms, effective echo time = 13 ms, field of view = 3 cm, matrix size = 256 x 256, slice thickness = 300 μm, number of averages = 2, axial (transverse) image orientation, and scan time = 6 min (Vahid-Ansari and Albert, 2018). Following MRI the animals were placed in their home cage and observed until recovery from anesthesia and then returned to the housing room.
C) Post-stroke anxiety phenotype in Pet-ChR2 mice. Pet-ChR2 mice (n=12) were tested for basal anxiety phenotype using elevated plus maze (EPM) one day before surgery (pre-) and 7 days post surgery (post-). The stroke mice (n=9) showed significantly lower duration in the open arm post-stroke compared to prior to surgery, while no difference was seen in Sham ctrl (n=3, data not shown). Data represent mean ± SEM, ****p* <0.001 by paired t-test.

Supplemental Figure 3

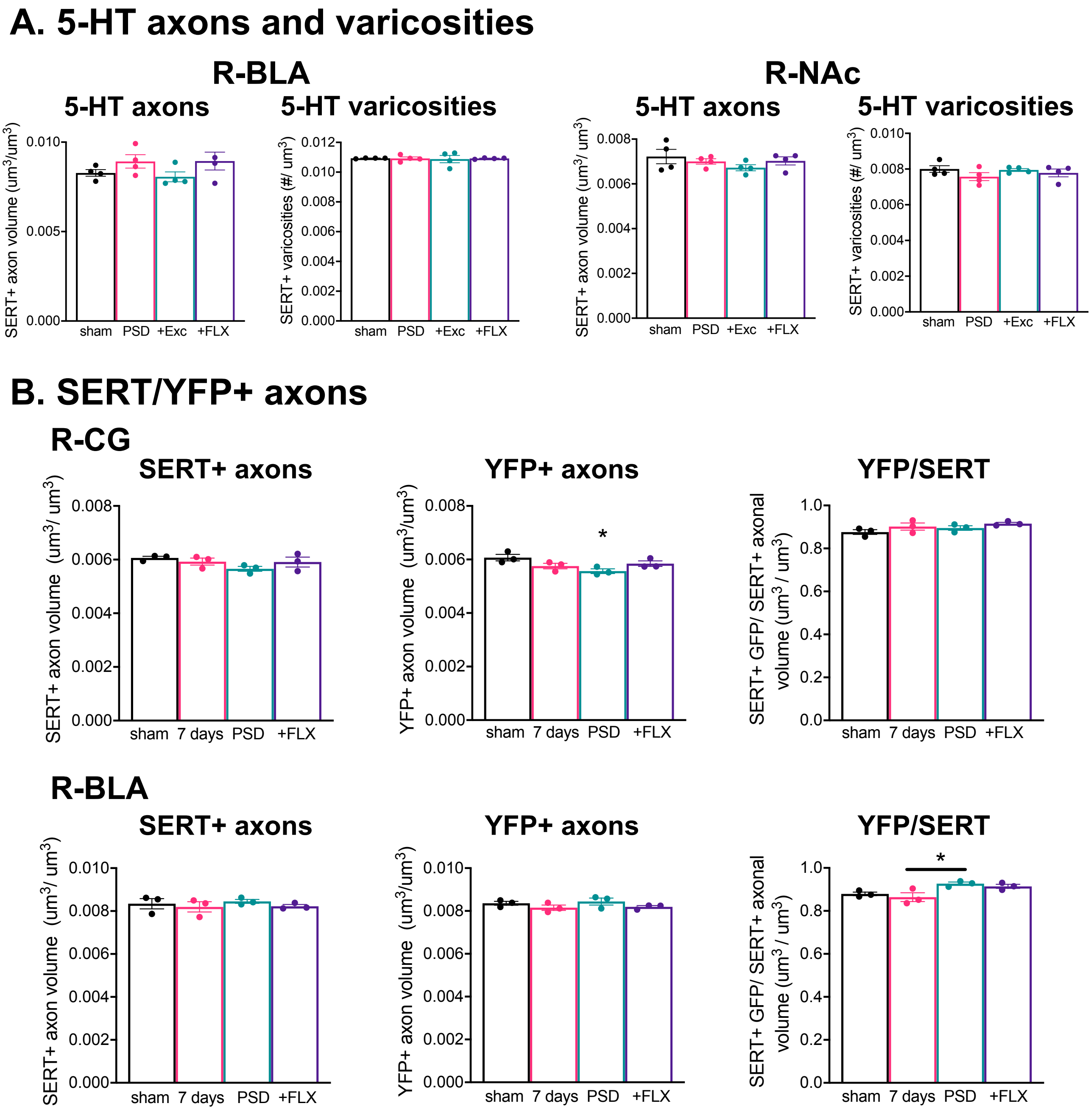

Supplemental Figure 3. Contralesional changes in SERT or YFP staining.

A. 5-HT axons and varicosities. Quantification of SERT-stained axonal and varicosity density is shown for the contralesional (right, R) basolateral amygdala (R-BLA) and nucleus accumbens (R-NAc) of Sham control, PSD vehicle, +Exc and +FLX mice (n=4). B, SERT/YFP+ axons. Sections of right CG and BLA from sham control (6 wks), 7 days post-stroke, 6 wks post-stroke treated with vehicle (PSD) or FLX (+FLX). Shown is quantification of SERT- and YFP-stained axonal density, and the ratio of YFP-SERT costained/total SERT+ axons of Sham control, PSD vehicle, +Exc and +FLX mice (n=3). Data are shown as mean ± SEM; *p<0.05 vs. sham or as indicated compared using one-way analysis of variance (Tukey's post-hoc).

Supplemental Figure 4

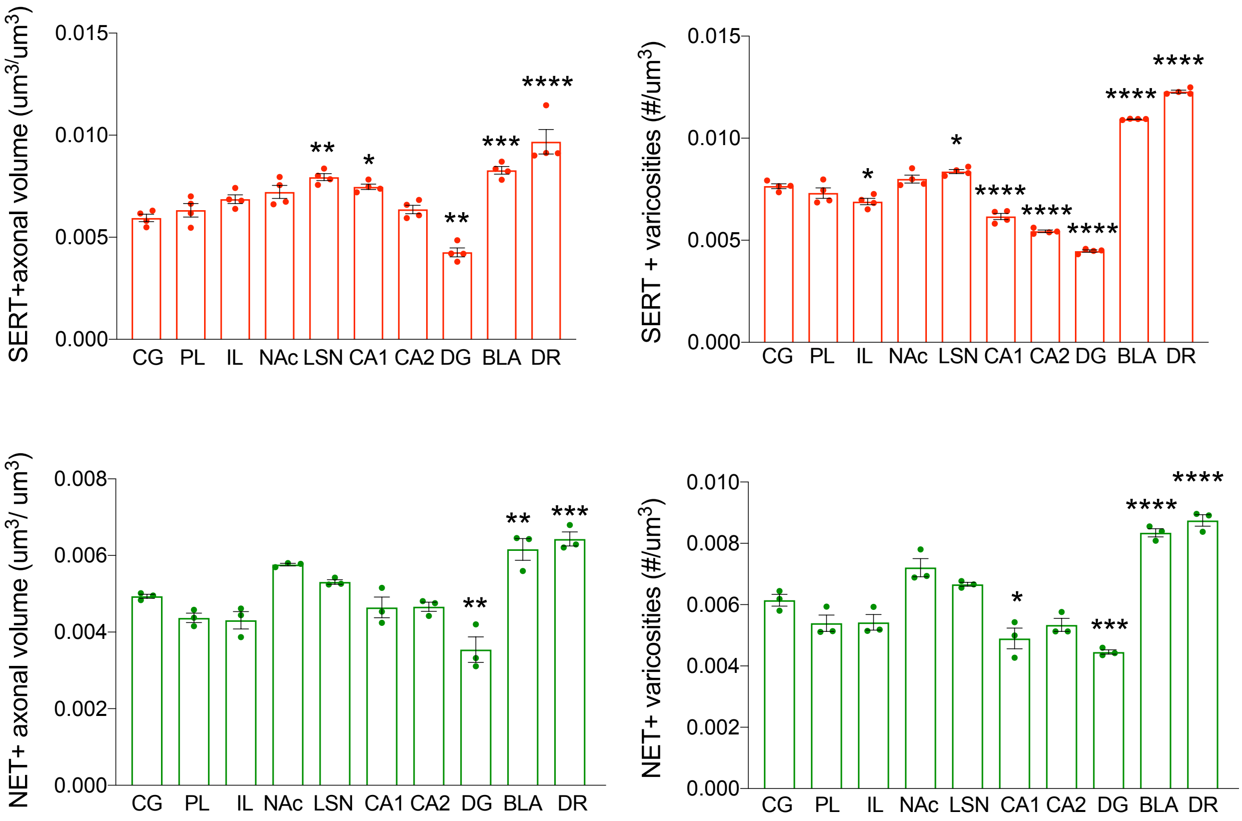

Supplemental Figure 4. Distribution of 5-HT and NE projections in mouse brain.

The density of SERT+ (above, n=4) and NET+ (bottom) axonal volume (right) and varicosity density (right) was quantified as described in Methods in brain regions of interest including: mPFC regions cingulate gyrus CG, prelimbic PL, and infralimbic IL; nucleus accumbens, NAc; lateral septal nucleus, LSN; hippocampal regions CA1, CA2/3, and dentate gyrus DG; basolateral amygdala, BLA; and dorsal raphe, DR. Shown are data from right side of sham mice, mean ± S.E. Shown are comparisons to CG, *p<0.05; **p<0.01; ***p<0.001, ***p<0.0001 one-way analysis of variance (Tukey's post-hoc).

Supplemental Figure 5

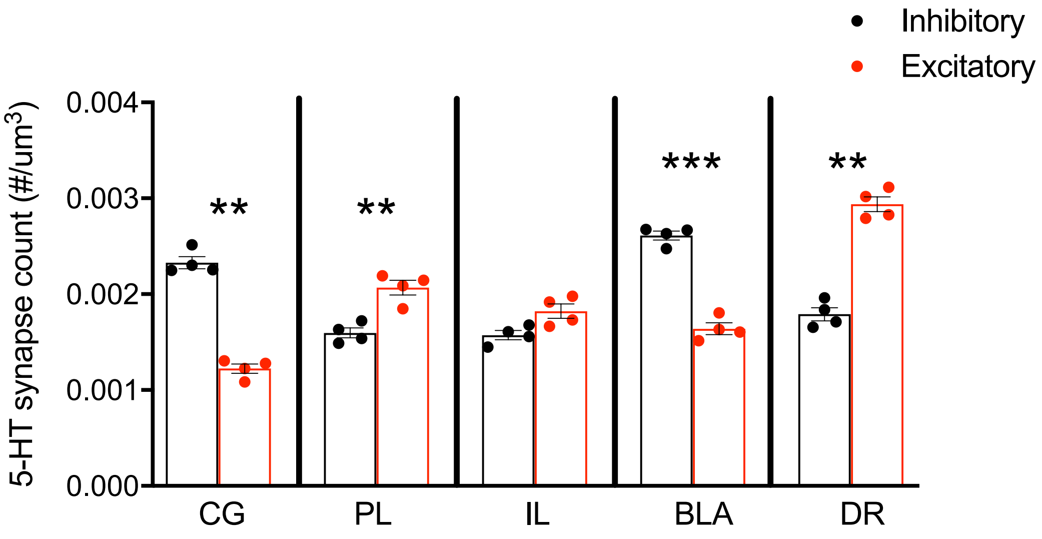

Supplemental Figure 5. Comparison of 5-HT synapses to inhibitory or excitatory synapses.

The number of 5-HT synapses (SERT/synaptophysin+) to inhibitory (gephyrin+) or excitatory (PSD95+) synapses is compared in brain regions of interest including: mPFC regions cingulate gyrus CG, prelimbic PL, and infralimbic IL; basolateral amygdala, BLA; and dorsal raphe, DR. Inhibitory and excitatory synapse counts from right side of sham mice are plotted as mean ± S.E. (n = 4) and compared using paired t-test; **p<0.01; ***p<0.001.

Supplemental Figure 6

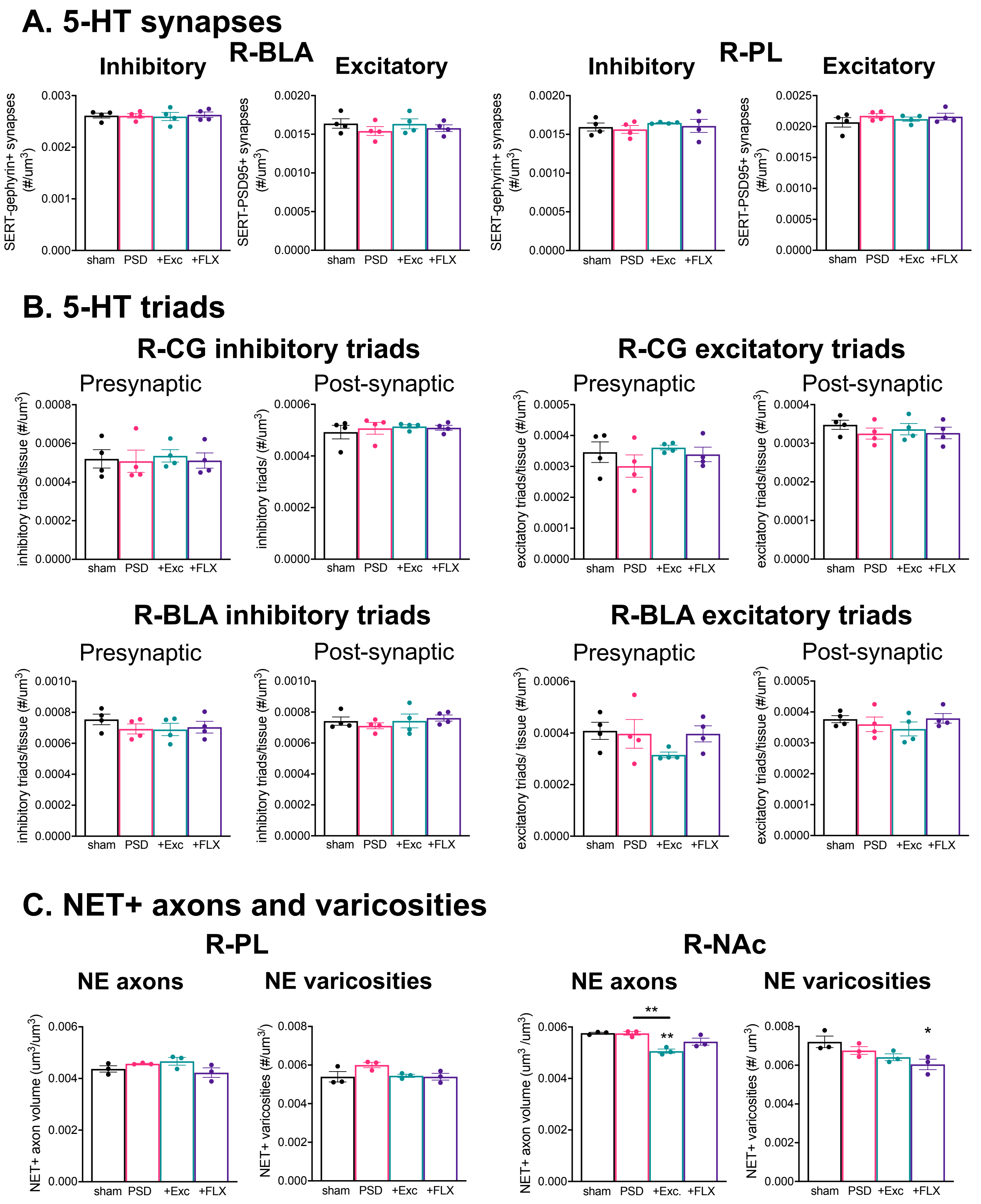

Supplemental Figure 6. Contralesional changes in 5-HT synapses and NE projections.

A. 5-HT synapses. Sections of right cingulate gyrus (CG) and prelimbic cortex (PL) were stained for SERT, synaptophysin and either PSD95 (5-HT excitatory synapses triads) or gephyrin (5-HT inhibitory synapses or triads). Quantification is shown for sections of Sham control, PSD vehicle, +Exc and +FLX mice (n=4). B. 5-HT triads. Sections from right CG and PL were stained for pre- and post-synaptic inhibitory and excitatory triads (see **Fig. 4**). Quantification is shown for sections of Sham control, PSD vehicle, +Exc and +FLX mice (n=4). C. NE axons and varicosities. NET+ axonal volume and varicosity density in right PL and nucleus accumbens (NAc) from sham, stroke treated with vehicle (PSD), exercise (+Exc) or FLX (+FLX) (n=3). Data are shown as mean ± SEM; *$p<0.05$, **$p<0.01$ vs. sham or as indicated compared using one-way analysis of variance (Tukey's post-hoc).
